# A Systematic Review of the Medical Student Feedback on Undergraduate Surgical Education During the Pandemic

**DOI:** 10.7759/cureus.30440

**Published:** 2022-10-18

**Authors:** Abdel Saed

**Affiliations:** 1 Orthopaedics, Barts Health NHS Trust, London, GBR

**Keywords:** teaching feedback, augmented reality (ar), simulated training, online teaching, virtual education

## Abstract

The coronavirus disease 2019 (COVID-19) pandemic has challenged and changed significant aspects of day-to-day life. With regard to medical education, the challenges have been substantial, and the changes have been innovative. This systematic review focuses specifically on medical student feedback on undergraduate surgical education during the pandemic. It explores the various types of technology used to facilitate online learning and aims to comprehensively review the advantages and disadvantages. The Preferred Reporting Items for Systematic Reviews and Meta-Analyses (PRISMA) guidelines were adhered to, and electronic databases PubMed, Medline, and Scopus were used to identify relevant studies. The search yielded 102 papers once duplicates and non-English articles were removed. Of these, 19 articles were included in the review. These publications were appraised, which was the source of the narrative syntheses of this systematic review, and due to the heterogeneous data, a meta-analysis could not be successfully implemented. The integration of real-time image capture devices used to display stakeholders or objects such as models of wounds has resulted in the improvement of virtual learning to an almost in-person experience. Adding to this, the use of communication and participation platforms facilitates active discussion when used appropriately. However, there are still some barriers that may be removed with time as the technology continually improves, and these are not exclusive to connectivity issues and restriction of the senses to only two-dimensional sight and hearing.

Despite this, the student feedback was largely positive, and the integration of more innovative methods of delivering teaching will have a positive impact on education as long as it is used as an adjunct and not as a replacement for face-to-face teaching.

## Introduction and background

The importance of undergraduate surgical education

Undergraduate medical education is designed to enable future doctors to attain the knowledge and skills needed to ensure they are competent junior doctors. Upon graduating, junior doctors are expected to be able to contribute to the safe care of patients [1]. However, undergraduate surgical education specifically is more complex due to the practical and labour-intensive requirements of the experience. The website of the Royal College of Surgeons England has useful aids for surgeons who will be teaching medical students. It provides a more uniform curriculum so that all students attain the same core competencies irrespective of the institution. These can be found for each surgical subspeciality [2].

Perioperative care should be covered in its entirety. This is important for aspiring surgeons and future general practitioners and physicians who are likely to manage patients during the pre-operative workup of patients as well as in their post-operative recovery and beyond [3]. The need for multidisciplinary team involvement and effective communication between the different specialities is evident. Surgeons and anaesthetists should be in regular dialogue with general practitioners regarding patients who are due to undergo surgery and require optimisation of chronic or acute conditions that may have adverse impacts on prognosis [4].

This is specifically laid out in the General Medical Council (GMC) of the United Kingdom’s agreed-upon "outcomes for graduates". This states that students who graduate must show competency in diagnosing, investigating, and managing clinical presentations across the community and in secondary care [5].

This is especially important as most newly qualified doctors in the United Kingdom rotate through surgical specialities [6]. Therefore, they need to be prepared for practice [7]. However, the learning needs are typically not comprehensively addressed. This is believed to be largely due to the gap between the theory taught and the limitations in practical, experiential learning [3]. Ultimately, this means that new graduates are unfamiliar with the all-encompassing clinical knowledge and skills required in treating patients with surgical conditions. As a result, newly qualified doctors have reported that they feel they are not as well prepared to manage emergency surgical on-calls and surgical placements as they are in dealing with medical placements and on-calls, even prior to the outbreak of the pandemic [8].

Aims and objectives

The aims and objectives of the systematic review were to review all appropriate current feedback regarding students’ impressions of undergraduate surgical education during the coronavirus disease 2019 (COVID-19) pandemic, both from qualitative and quantitative forms. The aims were as follows: (i) review innovative teaching delivery implemented by UK medical schools/universities during the pandemic to ensure the GMC graduate outcomes and similar pre-pandemic student experiences were minimally derailed; (ii) review students’ experience of these innovative teaching delivery methods; (iii) review whether undergraduate surgical education will likely progress with the use of technology discussed in this review.

Scope and limitations

This systematic review’s scope will focus on undergraduate medical students of any year group who are subjected to surgical education modules. Since this is not an original study, we will follow strict inclusion and exclusion criteria to ensure appropriate studies are included. There will be no limitation to population size, geographical location, or social demographics. Any study which has qualitative or quantitative feedback will be analysed and included.

## Review

Search strategy

The purpose of this search was to identify all eligible studies featuring the impact of COVID-19 on undergraduate surgical education. The present systematic review was performed in accordance with the Preferred Reporting Items for Systematic Reviews and Meta-Analyses (PRISMA) guidelines. In June 2022, a comprehensive search was conducted for all non-grey literature published using the online platforms PubMed, MEDLINE, and Scopus. No similar articles were found in the Cochrane Library.

The following search terms were used to ensure all appropriate studies were captured: "COVID-19" or "coronavirus*" or "2019-nCoV*" or "SARS-CoV-2" or "COV-19" or "outbreak" or "pandemic" or "novel coronavirus" and "surgical" and "education or curricul*" and "undergrad" and "virtual learning" or "online learning" or "e-learning" or "remote learning" or "distance learning" or "blended learning" or "electronic learning" or "teaching" or "online teaching". In addition, all relevant studies published up to and including June 2022 discussing the impact of COVID-19 on undergraduate surgical education were included. There was no dedicated publication time limitation as such, but by definition, as COVID-19 began in late 2019, all studies were limited to 2019 onwards. We further supplemented our search by exploring the reference list of all the included articles for additional eligible studies.

Inclusion and exclusion criteria

The aim was to include all published articles that discussed the impact of the COVID-19 pandemic on undergraduate surgical education. All study designs were retained due to the limited number of meta-analyses and the lack of historical data. In addition, due to the limited number of papers, all papers were subject to a full-text review. This included randomised controlled trials, cohort studies, case-control studies, case series, and case reports. Population, Intervention, Comparison, and Outcome (PICO) criteria were further specified to determine certain inclusion and exclusion criteria. The population was specifically medical students. All other studies focused on physicians, non-medical students, and school education reports, and other healthcare professionals such as dentistry, nursing, or veterinary were excluded. The intervention included innovations in surgical education used to mitigate the COVID-19 pandemic. All prospective or retrospective studies, non-randomised comparison studies, and case series were considered for inclusion. If more than one study was conducted at the same institution, the article with the most complete or recent data was selected. Basic science or animal studies, expert opinions, and grey literature were excluded.

Article selection

**Figure 1:**
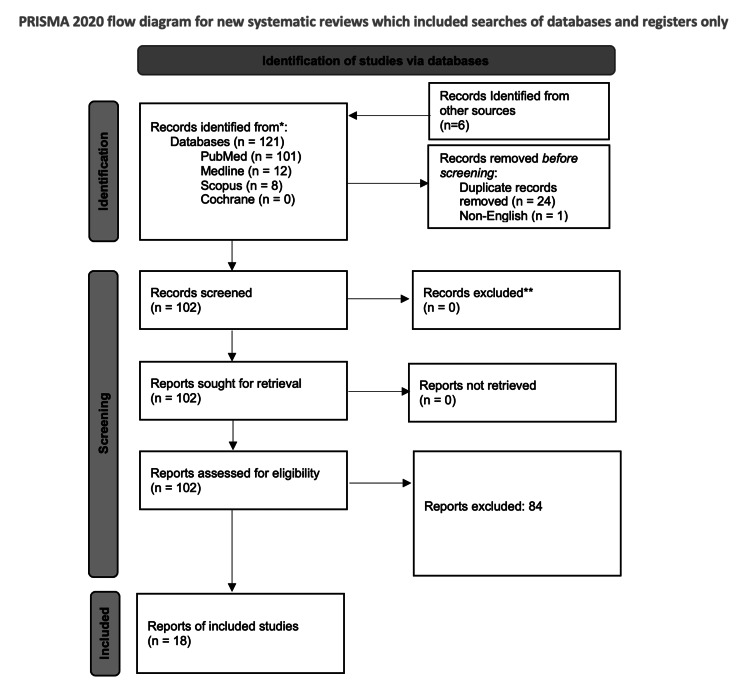
PRISMA flow diagram PRISMA: Preferred Reporting Items for Systematic Reviews and Meta-Analyses.

All potentially relevant articles were identified via the search strategy, and no further pertinent studies were identified when references were reviewed. Following the exclusion of duplicates, a total of 96 articles were included, and an additional six were identified that were not highlighted in the original search, totalling 112. All studies were subject to full-text review by the author, which led to 18 studies being included in this systematic review (Figure 1).

Results

Participants in the studies reviewed included 1,529 medical students and addressed various areas of pedagogy such as synchronous and asynchronous online teaching, blended teaching, live virtual shadowing, and augmented reality (AR) teaching. The number of students participating in each study was between six and 763. One study did not disclose the number of students involved; however, it was included due to the impactful feedback. The next section will discuss the 18 relevant studies to assess the impact of COVID-19 on undergraduate surgical education as seen in Figure 1.

Online Teaching

Most of the studies that will be discussed contain a mixture of synchronous virtual teaching and asynchronous internet-based teaching. However, for the benefit of this review, the author will encompass these forms of delivery of pedagogy as "Online Teaching". This will comprise all virtual teaching that does not include live shadowing clinical environments or patient encounters.

In a study by Pettitt-Schieber et al., when reviewing students' understanding of the subspeciality surgical course, a four-point Likert scale found a post-course score of 3.3 +/- 0.5 vs. a pre-course score of 2.0 +/- 0.8. All sessions were held over Zoom (Zoom Video Communications, Inc., San Jose, CA). Feedback in qualitative and quantitative forms was collected following the synthesis of questionnaires by each speciality. Eighteen virtual surgical electives (VSEs) were carried out with a minimum of two iterations of each surgical speciality. Out of the 67 students who filled out feedback forms, 67.2% and 25.4% reported feeling "very comfortable" and "comfortable", respectively, when using the Zoom videoconferencing software. In addition, 98.5% felt the course objectives were met either "very well" or "well" [9].

Schmitz et al.’s study had an experimental group that was required to utilise online platforms, while the control group received book chapters related to these specific anatomical regions. An interactive platform was synthesised for the online material to enable the teaching of operative techniques and skills. After examination, the students in the video group scored a higher percentage of correct answers (0.67 vs. 0.60) [10].

Chandrasinghe et al.’s study recruited 754 students via Facebook for an online teaching session [11]. Junior medical students presented the basic science on specified topics, while more senior medical students discussed a clinical case. Over 98% of the respondents felt that the discussions improved their clinical understanding. Also, 96% scored 4 or above (out of 5) on the question of how well they felt the sessions ran [11].

Shin et al. found that virtual case-based discussions improved medical students’ confidence in independently conducting initial assessments for surgical patients. For example, 16 students would each take a history and orally request examination findings from the tutor who acted as the patient. This was highlighted by a pre-course and post-course understanding with a Likert score of 2 and 4 out of 5, respectively [12].

At the Emory University School of Medicine in Atlanta, Georgia, a two-week VSE that involved direct interaction with the surgical faculty and self-directed learning was created. This involved didactic synchronous and asynchronous methods of teaching and a skills lab facilitated by the Zoom videoconference app to aid in the teaching of basic surgical skills. Of the 14 participating medical students, 91% felt the course met their learning needs “very well” or “well”. Pre-course and post-course understanding scores highlighted that 27% reported a “good” understanding of general surgery, and 100% reported either a “good” or “very good” understanding, respectively. In addition, 82% reported increased interest in general surgery [13]. The same institution synthesised a one-week virtual urology course, which consisted of interactive lectures, case-based discussions, and surgical reviews conducted via video. All nine medical students reported an increased understanding of the common urological conditions by an average of 2.5 points on a 10-point Likert scale. The majority of the students (56%) also responded by stating they had an increased interest in urology, while 22% reported a decreased interest [14].

Williams et al. conducted a study in Philadelphia, USA. They enrolled 10 senior medical students who undertook a two-week synchronous and asynchronous virtual urological surgery clinical rotation. This included pre-recorded lectures, video content, self-directed problem-based learning modules, an online discussion board, and real-time case discussions via videoconferences to name a few.

Median Likert scores out of five pre-course and post-course were as follows for each domain: overall knowledge (pre-course = 3 and post-course = 4); naming urological conditions: (pre-course = 2 and post-course = 4.5); urological evaluation confidence (pre-course = 2 and post-course = 3.5); urology consult confidence (pre-course = 3 and post-course = 5) [15].

Pang et al. conducted a study in the USA analysing the students' perspectives on a virtually informed consent activity. The majority of students stated they felt their ability was satisfactory or above on completion of the module [16].

A study by Newcomb et al. reviewed six medical students who attended a two-hour virtual class designed to improve their communication and rapport-building skills through video platforms. As an outcome, four out of the five student participants graded the class as "A+" [17].

A study by McGann et al. with 60 students responding to the feedback on an online basic surgical skills course they attended revealed that 83.7% felt the teaching was satisfactory, and the course either met or exceeded their expectations [18].

A study by Quaranto et al. on interactive remote basic surgical skills sessions found an improvement in the 31 participating medical students’ confidence scores in suturing and knot tying. Knot tying and suturing improved on completion of the course from 7.9 to 9.7/18 and 8.0 to 13.8/30, respectively [19].

A survey in India looking into students’ feedback regarding their online teaching experience yielded some adverse results. A total of 389 students completed the questionnaire, and 71.98% felt that the overall online classrooms adversely affected their learning. In addition, 93.32% felt their practical learning suffered, and 60.93% felt their theory learning was adversely affected [20].

In Co et al.’s study, before the pandemic, 30 final-year medical students were taught basic surgical skills face-to-face. The same group was then invited to attend an online web-based surgical skills learning (WSSL) session via Zoom with the same tutor, and the feedback was evaluated via standardised questionnaires [21]. The result indicated that 73.4% of the students felt that learning and demonstrating surgical knot-tying WSSL was no more difficult or easier than the face-to-face session. Of the students, 10% felt that WSSL was easier to follow than the face-to-face sessions. Of the students, 40% highly recommended WSSL with a score of 9 or greater out of 10, while 50% gave a score of 6-8 out of 10 [21].

Blended Teaching

Blended teaching involves integrating traditional tutor-led classroom activities with technology [22]. Lindeman et al. studied 29 participants’ impressions of blended learning. Feedback regarding the blended course and face-to-face teaching using a five-point Likert scale was 3.80 vs. 3.52 for the lecture series. Teaching effectiveness was 4.30 vs. 3.93 [22].

Live Virtual Shadowing

This section will cover studies that discuss the use of technology in the live clinical environment. In Byrnes et al.'s study, a two-week virtual elective was offered to medical students at the University of Pennsylvania in which six participated [23]. The virtual elective is comprised of the following three major components:

Virtual operating room (OR): The attending surgeon would wear a head-mounted GoPro camera (GoPro, Inc., San Mateo, CA) allowing students to watch the procedures and communicate with the surgeon.

Telehealth: It allowed surgeons to have students join them when conducting video conferences with patients. Students could conduct the initial consultation with the patient and then report back to the surgeon.

Virtual didactics: Students would present patients at the virtual multidisciplinary head and neck tumour board.

On the five-point Likert scale, the average student rating of the telehealth sessions was 4.2, the virtual operating room was 4.0, and the overall virtual didactics was 4.5 [23].

At the same institution, a virtual otolaryngology surgery rotation, which comprised livestream interactive surgeries, virtually run small group didactics, and outpatient telehealth visits were synthesised. The findings were that the virtual elective was not a suitable replacement for a true experience in the clinical environment. However, students responded that they felt the virtual week gave them more one-on-one time with senior surgeons compared to traditional electives and that they could see more of the operation than if they were in the operating theatre [24].

Across the USA, the Vanderbilt Otolaryngology online medical student experience was a virtual course that enabled online grand rounds, teaching led by residents, and simulated "on-call" sessions. The average Likert score out of 5 was 4.05 for demonstrating interest and 4.62 for supporting students during the pandemic. Demonstrating average knowledge score was 3.57 [7].

Augmented Reality Teaching

Augmented learning is a learning medium in which the environment adapts to the learner [25]. Luck et al. used the HoloLens headset (Microsoft Corporation, Redmond, WA) that utilises a mixed reality optic display capability to supplement a "surgeon’s eyes", allowing the 60 student participants in a series of remotely-delivered simulated ward rounds.

Feedback came from 47 students. Of the respondents, 90% "agreed" or "strongly agreed" that AR could improve undergraduate surgical training. They recommended and would like to see the HoloLens AR workshop continue post-COVID-19 pandemic. Furthermore, 85% of students responded that they enjoyed the AR workshop (Table 1) [25].

**Table 1:** Summary of studies included VSE: virtual surgical elective; WSSL: web-based surgical skills learning.

Articles	Location	Sample size (N)	Study duration (weeks)	Study aim	Study outcome
Online teaching					
Pettitt-Schieber et al. (2021) [9]	USA	67	3.5	Evaluation of the 8 VSEs	Learning needs met (98/5%); comfort with technology (92.6%); student understanding pre-course (2/5), post-course (3.3/5)
Schmitz et al. (2021) [10]	Undisclosed	58	Undisclosed	Development of a tailor‐made surgical online learning platform, ensuring surgical education in times of the COVID-19 pandemic	Post-course test scores of controls vs. online learning platform users (0.60 vs. 0.67)
Chandrasinghe et al. (2020) [11]	Sri Lanka	754	6	Online teaching to improve understanding of general surgery for medical students	Over 98% of the respondents felt that the discussions improved their clinical understanding. 96% scored 4 or above (out of 5) on the question of how well they felt the sessions ran
Shin et al. (2021) [12]	USA	16	8	Effects of virtual case-based discussions on medical student understanding	Pre-course understanding, Likert score of 2 out of 5, compared with a post-course score of 4 out of 5
Grady et al. (2022) [13]	USA	14	2	Effects of the VSE, which involved synchronous and asynchronous teaching and skills labs – basic surgical skills	91% felt the course met their learning needs "very well" or "well". Pre-course and post-course combined "good" and "very good" understanding – 27% vs. 100%
Kuo et al. (2021) [26]	USA	7	1	Efficacy of vascular virtual medical student education during the coronavirus disease 2019 pandemic	Improved results in pre- and post-course assessments
Manalo et al. (2021) [14]	USA	9	1	Understanding following a virtual urology course, which consisted of interactive lectures, case-based discussions, and surgical reviews conducted via video	Increased understanding of the common urological conditions by an average of 2.5 points on a 10-point Likert scale
Williams et al. (2021) [15]	USA	10	2	Feedback following a virtual urological surgery clinical rotation	Overall knowledge: pre-course (3), post-course (4). Naming urological conditions: pre-course (2), post-course (4.5). Urological evaluation confidence: pre-course (2), post-course (3.5). Urology consult confidence: pre-course (3), post-course (5)
Pang et al. (2021) [16]	USA	Undisclosed	34	Feedback of a virtually informed consent activity	The majority of students felt their ability was satisfactory or above on completion of the course
Newcomb et al. (2021) [17]	USA	6	Undisclosed	To enable students to improve their communication and rapport-building skills through video	4 out of the 5 student participants graded the class as "A+" highlighting that the session not only taught them new skills but also reinforced existing ones
McGann et al. (2021) [18]	USA	60	Undisclosed	The benefit undergraduate medical students gained from an online basic surgical skills course	83.7% regarded the impact of the teaching as satisfactory and students felt the course either met or exceeded their expectations
Quaranto et al. (2021) [19]	USA	31	Undisclosed	Impact of interactive remote sessions on surgical instruments, suture techniques, and knot tying	Knot tying improved from 7.9 to 9.7/18 whilst suturing improved from 8.0 to 13.8/30
Ray et al. (2021) [20]	India	389	12	Medical student perspective regarding undergraduate surgical training	71.98% felt that the overall online classrooms adversely affected their learning. In addition, 93.32% felt their practical learning suffered, and 60.93% felt their theory learning was adversely affected
Co et al. (2020) [21]	Hong Kong	30	1	Face-to-face surgical skills teaching vs. online web-based teaching	73.4% of the students felt that learning and demonstrating surgical knot-tying WSSL was no more difficult or easier than the face-to-face session. 10% of students felt that WSSL was easier to follow than the face-to-face sessions

Blend teaching					
Lindeman et al. (2015) (22]	USA	29	8	Feedback regarding the blended course and face-to-face teaching	3.80 vs. 3.52 for the lecture series. Teaching effectiveness was 4.30 vs. 3.93, respectively (using a 5-point Likert scale)
Virtual live shadowing					
Byrnes et al. (2021) [23]	USA	6	2	Student ratings of the virtual operating room, virtual didactics, and telehealth	The average student rating of the telehealth sessions was 4.2, the virtual operating room was 4.0, and the overall virtual didactics was 4.5
Chao et al. (2021) [24]	USA	Undisclosed	Undisclosed	Feedback following virtual otolaryngology surgery rotation, which comprised livestream interactive surgeries, virtually run small group didactics and outpatient telehealth visits	Students felt the virtual week gave them more one-on-one time with senior surgeons compared to traditional electives and that they could see more of the operation than if they were in the operating theatre
Augmented reality teaching					
Luck et al. (2021) [25]	United Kingdom	47	Undisclosed	Feedback regarding the Hololens headset to supplement the "surgeon’s eyes"	90% of respondents "agreed" or "strongly agreed" that augmented reality could improve undergraduate surgical training

Discussion

There has been a drift from traditional didactic classroom teaching to a student-centred learning environment. As a result, the principles that guide education delivery have drastically changed over the past few decades [27]. Acquiring feedback from students involved in educational activities has become integral; hence, this systematic review only includes studies where student feedback was recorded.

Prior to the pandemic, synchronous distant education (SDE) was used widely in varied health science cohorts with higher overall satisfaction compared to traditional education [28].

On reviewing the literature, it is clear to see that existing teaching and learning technologies, which include hardware and software in many institutions, were enhanced in an attempt to mitigate the negative impacts the COVID-19 pandemic was having on undergraduate surgical education.

Distance learning delivered online can typically be done in two formats: asynchronous and synchronous. Asynchronous involves techniques such as recorded videos, podcasts, and other miscellaneous e-learning content accessible to students at any time. In contrast, synchronous teaching involves, but is not limited to, live virtual classrooms and video conferences [29].

A combination of both synchronous and asynchronous pedagogy is termed the flipped classroom. This allows both the benefits of interaction in synchronous and the flexibility of asynchronous to be experienced by the students [1]. The author's view is derived from the literature and experiential learning. This variation is one of the keys to maximising educational performance. Varying the delivery method of the teaching content and adding new innovative media is of value to students, as long as it is implemented effectively. This view is supported by the positive feedback from the studies discussed in this review and the single study that yielded adverse feedback regarding the use of distant-based learning. When the adverse outcomes from Ray et al.’s study were critiqued, the reason was poor implementation [20].

Four specific types of technology, i.e., online teaching, blended teaching, live virtual shadowing, and AR teaching, used to mitigate the lost face-to-face learning time were reviewed. These pedagogy methods fall into the online distance education (ODE) category. ODE allows greater flexibility with location and time, increasing convenience for all involved stakeholders [29]. ODE’s cost-effectiveness compared to classroom-based learning is also noteworthy [29]. This type of teaching may not only help bridge a deficit but also provide an opportunity for improved learning away from traditional teaching environments. Being able to replace certain aspects of the curriculum with asynchronous, readily available teaching material, which students can access at their convenience, is advantageous for all stakeholders. Pre-recorded videos, if created appropriately and comprehensively, at most may need periodical updates [30]. Online learning assists students in becoming familiar with the inevitable transition into the web-based medical world and the digital health technology that will be more prevalent as time passes [29]. Regarding digital health technology, research has shown comparable clinical outcomes found in person and telehealth post-operative visits [17]. Therefore, if it is integrated into surgical care, students must be exposed to and familiar with the technology to ensure they are competent when graduating.

The student feedback regarding the variations of online teaching they received has been largely positive. Population sizes varied between six and 754. Outcome measures were varied and subjective. However, the most commonly assessed outcome measure was pre- and post-course "understanding" using either a five- or 10-point Likert scale. All but one of the studies have shown a statistically significant improvement in all the domains in which they have collected feedback. It is clear that outcomes cannot be solely attributed to pedagogy reliably. Other factors include but are not exclusive to the facilitator not being experienced or well prepared, the content of the teaching not being at an optimum and appropriate level, the teaching not meeting the learning outcomes, and so on. The need to teach particular video-based communication skills to ensure future clinicians have the necessary skills to build rapport with patients and their next of kin is evident. This is especially important in this commonly two-dimensional distant communication method. In an interview with Association of American Medical Colleges, Neal Sikka, MD, discussed the evolving need for telemedicine training in medical school. He stated that "there really is an art to providing a good video consultation that needs to be taught, just like we teach bedside manner and patient interviewing skills" [17]. Students are required to learn how to be empathetic and show attention when separated from patients by a video screen. The lack of direct eye contact and appropriate physical contact can be mitigated to a degree by increased vocalisation of empathy and other appropriate verbalised emotional responses. Experts in medical education have aptly named this "digital empathy", and there has been a recommendation to include this in the undergraduate curriculum along with the advised increased practice in telemedicine [31].

On the whole, the general perception of medical students who received online surgical education was that they attained both what they needed and wanted [32]. However, for students to be able to make an informed decision about whether or not they want to pursue a career in a specific speciality, they need to discern whether the daily work of a said speciality fits in with the student’s professional and personal aspirations. Distance learning is limited in this aspect of education [24].

Undergraduate surgical education is more challenging for inexperienced medical students with access to delicate clinical environments where highly specialised skills are practised while keeping patients safe. Achieving this throughout an entire cohort of medical students makes this even more challenging. Distance learning may provide a means of improving this difficult situation, although it cannot be considered a total replacement. Therefore, a continually thorough and thoughtful evaluation of the efforts made by various institutions during the pandemic is necessary and is the only way serial improvement in medical education will progress.

Limitations of the study

It is also important to note that in the studies included, only one study by Schmitz et al. had controls, and another study by Co et al. subjected the same group of students to the traditional face-to-face and online methods. This impacts the effectiveness of the online teaching method, which needs to be considered as the students have already been exposed to the teaching material and their baseline understanding had been altered. One could therefore argue that the results from this study could be met with caution. The remaining studies were observational in nature.

Meta-analysis could not be performed due to the heterogeneity of data as there are many other variables that could not be reliably controlled. These include but are not exclusive to institutions, the technology available, and student engagement/receptiveness to the technology offered.

Recommendations

Successful execution of remote learning courses needs significant technological input. All systems and software must be optimised, maintained, and function at both ends for the students and the teachers. This is even more sensitive when the teaching is synchronous.

With regard to video conferencing software, teachers may default to the technology available at the institution. However, it is also imperative that the students can install the software on their devices.

Regular feedback from all stakeholders, including the facilitators, is important to ensure the continual development of the teaching session.

## Conclusions

It is without a doubt that the COVID-19 outbreak brought unforeseen challenges in the field of surgical undergraduate medical education and subsequently accelerated the development of distant teaching methods. This review demonstrates that technology has been implemented with largely positive results. We are now moving out of the pandemic restrictions and learning how to "live with COVID-19". It is clear that the accelerated use of technology has helped mitigate the lack of contact teaching, and there is scope for it to continue to be helpful in the future. The literature has demonstrated that we can make the agile change in many areas of undergraduate surgical education. The future of undergraduate surgical education should continue to be agile, especially as technology advances. It should also be carefully implemented considering the limitations of the location. It can be infrastructural issues or variations in societal demands in the country in which it is implemented. However, as tempting as it may be, these platforms must be used as an aid and not a replacement for real-life experiences. The literature supports the concept of hybrid surgical education, in which virtual sessions and traditional face-to-face teaching are implemented. If implemented effectively, virtual teaching can provide students with foundational theoretical knowledge, which could be built upon during face-to-face teaching. Significant challenges must be considered in the continual development of online learning, and medical institutions must be prepared to utilise tech-based pedagogy to facilitate a successful online educational environment for medical students. Institutions must consider ways to continually support, advise, and motivate students and encourage and be receptive and reactive to feedback. This will support medical students in adapting to this type of pedagogy, which will likely become even more prevalent in the future.
